# Supplementary material for: Continuity in geometric intuition between humans and monkeys
Source: Proc Natl Acad Sci U S A. 2026 Jul 20;123(30):e2532934123. doi: 10.1073/pnas.2532934123 (PMC13416681; doi:10.1073/pnas.2532934123)
Supplement: Supplementary file 1 — Appendix 01 (PDF) [file pnas.2532934123.sapp.pdf]

Supporting Information for

Continuity in Geometric Intuition Between Humans and Monkeys

Jialin Li[+], Isabelle Boni[+], Logan R. Sandwick, Emily M. Sanford, Caroline M. DeLong, Li Wenjie, Margret M. Henderson, Steven T. Piantadosi[*], and Jessica F. Cantlon[*]

Steven T. Piantadosi & Jessica F. Cantlon.
E-mail: stp@berkeley.edu & jcantlon@andrew.cmu.edu

This PDF file includes:

Supporting text
Figs. S1 to S14
SI References

Supporting Information Text

Results

1. Group Accuracy in the Intruder Task and the Match-to-Sample Tasks.

All groups showed a significant above-chance accuracy which eliminated the confounding factor from learning. The Intruder task is the most difficult task (chance level = 1/6) while the chance level of Match-to-Sample Tasks are 1/2. Adults showed the highest accuracy across all tasks (the Intruder Task: [mean = 0.63, SE = 0.01], the Match-to-Sample Task: [mean = 0.93, SE = 0.00], the rotated Match-to-Sample Task: [mean = 0.96, SE = 0.01]), followed by preschoolers (the Intruder Task: [mean = 0.49, SE = 0.01], the Match-to-Sample Task: [mean =0.92, SE = 0.00] the rotated Match-to-Sample Task: [mean = 0.86, SE = 0.01]) and monkeys (the Intruder Task: [mean = 0.47, SE = 0.00], the Match-to-Sample Task: [mean = 0.75, SE = 0.00], the rotated Match-to-Sample Task: [mean = 0.79, SE = 0.00]).

2. Logistic Regression of IT and Symbolic

To identify which representations predicted behavior in non-human primates, preschoolers, and Tsimane' adults, we conducted logistic regression analyses using the glm package in R. For each trial, the representational distance between the sample shape and the left choice was subtracted from the distance between the sample shape and the right choice, providing a directional measure of how participants compared the two options. The outcome variable, choice of the right shape, was coded as binary: choice of the right shape will be 1, and the left shape will be 0. To avoid collinearity effects, each model included only one representation as the predictor. To provide more detail on the response functions, we fit a simple logistic regression, testing how three representations (V1, IT, Symbolic) predict behavior across groups in both version of Match-to-Sample Task. For the unrotated Match-to-Sample Task, V1 showed weak effects in all groups: monkeys (β = -0.17, $p < .001$) and preschoolers (β = -0.21, $p < .001$) and Tsimane' adults (β =1.14, $p < .001$). IT showed large positive effects in all groups: monkeys (β = 1.16, $p < .001$), preschoolers (β = 2.98, $p < .001$), and Tsimane' adults (β = 3.23, $p < .001$). Symbolic effects were positive and significant in all groups: monkeys (β = 1.14, $p < .001$) and preschoolers (β = 3.01, $p < .001$), and Tsimane' adults (β = 4.16, $p < .001$). Adults showed the strongest symbolic responses, followed by preschool children, and then monkeys. For the rotated Match-to-Sample Task, symbolic showed a larger positive effect in monkeys (β = 2.62, $p < .001$) and US adults (β = 9.46, $p < .001$) than unrotated Match-to-Sample Task, while the symbolic effect for preschoolers remains stable (β = 2.64, $p < .001$). IT effects were still significantly positive across all groups (monkeys: β = 3.48, $p < .001$; preschoolers: β =3.32, $p < .001$; US adults: β = 3.00, $p < .001$), while V1 kept the weak effect (monkeys: β = 0.19, $p < .001$; preschoolers: β = 0.16, $p < .001$; US adults: β = 0.52, $p < .001$).

3. The Regularity Effect across Groups and Tasks.

We asked whether the symbolic representation of geometric shapes, represented by the regularity effect, can explain the behavior of monkeys and humans in the Intruder Task and the Match-to-Sample Task. The regularity effect is indexed by the total number of regular features (e.g., equal sides, right angles, equal angles) contained by each shape, following Sablé-Meyer et al. (2021) (1). We calculated the relative distance in regularity as a predictor of accuracy on each trial. We used a linear mixed regression to test the regularity effect, predicting Accuracy from Regularity, Group, Task, their interactions, and a random effect of Subject. If the regularity effect is unique to humans, we should see that it only explains performance in human groups but not in monkeys. We found the opposite: The symbolic representation showed a significant main effect in all groups (β = 0.29, $p < .001$). There was a significant interaction between Regularity and Task (β = -0.22, $p <0.001$), suggesting that regularity had a greater effect in the Match-to-Sample Task than the Intruder Task. There was a significant interaction between the Regularity effect and Group, preschoolers and adults have stronger regularity effects than monkeys (preschooler: β = 0.04, $p < .001$, adults: β = 0.04, $p < .001$). The intercept was positive and highly significant (β = 0.91, $p < .001$), indicating the general above-chance baseline performance. The Intruder task was more difficult than the Match-to-Sample Task (β = –0.68, $p < .001$). Group comparisons showed that both preschoolers (β = 0.11, $p < .001$) and adults (β = 0.16, $p < .001$) performed significantly better than monkeys. The model explained 30% variance of the data (R^2 = 0.30).

4. Group-level Bayesian Analysis

A. Lapse Rate. We included the lapse rate as a parameter to quantify the noise in the behavior in the Bayesian regression model. The more random in the behavior, the closer the lapse rate to 1. All participants showed relatively low lapse rates (<0.3).

In the V1-IT-Symbolic model, preschoolers showed the highest lapse rate (the Intruder Task: mean = 0.16, HDI = 0 - 0.27; the Match-to-Sample Task: mean = 0.06, HDI = 0.04-0.08), followed by adults (the Intruder Task: mean = 0.08, HDI = 0 - 0.16; the Match-to-Sample Task: mean = 0.01, HDI = 0-0.01), and monkeys (the Intruder Task: mean = 0, HDI = 0 - 0.01; the Match-to-Sample Task: mean = 0, HDI = 0-0.01). The pattern of lapse rate of V1-IT-IT Rot Inv in the Intruder Task remains the same as the V1-IT-Symbolic model: preschoolers ranked the highest (mean = 0.29, HDI = 0.19 - 0.39), followed by monkeys (mean = 0.23, HDI = 0.19-0.27) and Himba adults (mean =0.28, HDI = 0.24 - 0.33). In the rotated Match-to-Sample Task, all groups showed a higher lapse rate in the V1-IT-Symbolic model (Monkeys: [mean = 0.07, HDI = 0.06-0.08]; preschoolers: [mean = 0.08, HDI = 0.04-0.11]; US adults: [mean = 0.01, HDI = 0-0.02]) than in the unrotated Match-to-Sample Task.

B. V1-IT-Symbolic Model. To understand how rotation changes the use of different geometric representations, we used V1, IT, and Symbolic predictors to model behavior in monkeys, preschoolers, and US adults. Figure S6 summarizes the Bayesian model weights for V1, IT, and Symbolic representations in monkeys, preschoolers, and US adults, as a supplemental figure for Figure 6. US adults ranked highest in symbolic representation [mean = 3.72, HDI = 3.14-4.29]. Preschoolers had higher symbolic scores [mean = 2.32, HDI = 1.62-3.00] than monkeys [mean = 1.40, HDI = 1.25-1.56]. IT representations were highest in monkeys [mean =3.85 , HDI = 3.15-1.54] and preschoolers [mean = 2.67, HDI = 1.77-3.48], while adults ranked the lowest [mean = 0.87, HDI = 0.30-1.47]. V1 representation did not contribute significantly in any group (US adults: [mean = 0.02, HDI = -0.39- 0.44], preschoolers [mean = -1.0, HDI = -0.49- 0.35], monkeys [mean = -0.26, HDI = -0.42- -0.11]).

C. V1-IT-IT Rot Inv-Symbolic Model. To further understand how the IT Rot Inv representation overlaps with the symbolic representation, we used a model that included the distance between shapes from V1, IT, IT Rot Inv, and Symbolic representations to predict behavior in monkeys, preschoolers, and Tsimane adults. If the IT Rot Inv representation overlaps with the symbolic representation, the symbolic weight should decrease when the model includes the IT Rot Inv. The more similar the IT Rot Inv representation and the Symbolic representation are, the more symbolic representation decreased.

Figure S7 summarizes the Bayesian model weights for V1, IT, IT Rot Inv, and Symbolic representation. The Symbolic representation decreased and is smaller but significant after accounting for the effect from IT Rot Inv (the Intruder Task: Himba adults: [mean = 0.90, HDI = 0.79 - 1.01], Preschoolers: [mean = 0.41, HDI = 0.28 - 0.53], Monkeys: [mean = 0.62, HDI = 0.60 - 0.64]; the Match-to-Sample Task: Monkeys: [mean = 0.75, HDI = 0.57 - 0.93], Preschoolers: [mean =1.32, HDI =0.20 - 2.35], US Adults: [mean = 1.77, HDI =0.66 -2.82]). The contribution of IT Rot Inv in humans behavior in the Intruder Task (Himba adults: [mean = 0.21, HDI = 0.11 - 0.31], Preschoolers: [mean = 0.23, HDI = 0.12 - 0.34]) is higher than monkeys (mean = 0.01, HDI = -0.02 - 0.03). All groups in the rotated Match-to-Sample task also showed the reliance on the IT Rot Inv (Monkeys: [mean = 1.37, HDI = 1.14-1.58], Preschoolers: [mean =1.98, HDI =1.14 - 2.90], US Adults: [mean = 3.29, HDI =2.35 - 4.30]). The contribution of IT is robust (the Intruder Task: Himba adults: [mean = 0.32, HDI = 0.21 - 0.43], Preschoolers: [mean = 0.41, HDI = 0.25 - 0.56], Monkeys: [mean = 0.37, HDI = 0.35 - 0.40]; the Match-to-Sample Task: Monkeys: [mean = 1.15, HDI = 0.80 - 1.45], Preschoolers: [mean =1.37, HDI = 0.29 - 2.40]) except US Adults in Rotated Match-to-Sample Task (mean = -0.17, HDI = -0.92-0.60). V1 still has little or negative contribution (the Intruder Task: Himba adults: [mean = 0.09, HDI = 0.01 - 0.17], Preschoolers: [mean = -0.03, HDI = -0.12 - 0.06], Monkeys: [mean = 0.16, HDI = 0.14 - 0.17]; the Match-to-Sample Task: Monkeys: [mean = -0.16, HDI = -0.28 - -0.05], Preschoolers: [mean =0.11, HDI =-0.44 - 0.66], US Adults: [mean = 0.11, HDI =-0.36 - 0.57]). Importantly, once Rotation Invariance was accounted for in the model, preschoolers' advantage over monkeys in Symbolic representation was eliminated.

5. Participant-level Bayesian Analysis

To test if our group-level Bayesian analysis result is driven by certain participants, we fitted the same model for each participant.

A. V1-IT-Symbolic Model. Figure S8 summarizes the participant-level Bayesian weights for V1, IT, and Symbolic model, which reaffirmed the results from the group-level Bayesian model. Monkeys, preschoolers, and adults showed a IT-Symbolic mixture strategy to represent the geometry in both the Intruder Task and the Match-to-Sample Task. Specifically, Adults showed the highest symbolic weight (the Intruder Task: mean = 1.43, SD =0.36; the Match-to-Sample Task: mean = 1.52, SD = 0.35), followed by preschoolers (the Intruder Task: mean = 1.02, SD =0.37; the Match-to-Sample Task: mean = 1.27, SD = 0.41), and monkeys (the Intruder Task: mean = 0.65, SD = 0.18; the Match-to-Sample Task: mean = 0.62, SD = 0.38). Human groups showed higher preference of IT representation (Himba adults: mean = 0.43, SD = 0.33; preschoolers: mean = 0.58, SD = 0.44) than monkeys (mean = 0.41, SD = 0.08) in the Intruder Task. Same pattern of IT representation was found in the Match-to-Sample Task (Tsimane adults: mean = 2.06, SD = 0.41; preschoolers: mean = 2.32, SD = 0.45, monkeys: mean =0.43, SD =0.43. In contrast, V1 representation was not favored by any participant groups: (the Intruder Task: Himba adults: mean = 0.22, SD = 0.28; preschoolers: mean = 0.05, SD = 0.34, monkeys: mean = 0.17, SD = 0.08; the Match-to-Sample Task: Tsimane adults: mean = 0.15, SD = 0.38; preschoolers: mean = -0.39, SD = 0.28, monkeys: mean = -0.16, SD = 0.16).

B. V1-IT-IT Rot Inv Model. Figure S9 plots the participant-level Bayesian weights for IT Rot Inv and Symbolic representation, which shows that the participant-level analysis matched the group-level analysis in the comparison of IT rotational invariant representation and the symbolic weight. Similar to our group-level analysis, human groups showed a higher weight of IT rotational invariant (Himba adults: mean = 1.41, SD = 0.39; Preschoolers: mean = 1.04, SD = 0.35) than Monkeys (mean = 0.53, SD = 0.18). IT also showed a robust contribution across groups (Himba adults: mean = 0.60, SD = 0.31; Preschoolers: mean = 0.68, SD = 0.44; Monkeys: mean = 0.68, SD = 0.16), while V1 showed a global, limited effect (Himba adults: mean = 0.13, SD = 0.24; Preschoolers: mean = 0.03, SD = 0.37; Monkeys: mean = 0.21, SD = 0.07).

C. V1-IT-Symbolic Model in Rotated and Normal Match-to-Sample Task. Figure S10 shows the participant-level Bayesian weights for V1, IT, Symbolic model in the Rotated Match-to-Sample Task (Monkeys and Preschoolers). When we compared the Bayesian weights between the monkeys with rotated geometric shapes and preschoolers with unrotated shapes, we found that the participant-level analysis converges with the group-level analysis. Monkeys with rotated shapes have higher symbolic weights than with unrotated shapes (Rotated: mean = 1.55, SD = 0.16, Unrotated: mean =0.62, SD = 0.38), and about the same level as preschoolers with unrotated shapes (preschoolers: mean =1.27, SD = 0.41). Similarly, preschoolers with rotated shapes have higher symbolic weight than with unrotated shapes (mean = 1.64, SD = 0.52).V1 and IT did not differ from the

group level analysis in rotated Match-to-Sample Task (Monkeys: V1: mean = -0.25, SD = 0.19; IT: mean = 2.52, SD = 0.89; Preschoolers:V1: mean = -0.02, SD = 0.20; IT: mean = 1.69, SD = 0.33).

Supplemental Discussion.

6. Key Differences in Approach from Sablé-Meyer et al. (2021)

Our analytic framework differs in several important respects from that of Sablé-Meyer et al. (2021)(1). These differences affect sensitivity to heterogeneous representations and the level of analysis at which behavior is modeled.

A. Trial-Level Bayesian Modeling vs. Aggregated Multiple Regression. Sablé-Meyer et al. (2021) evaluated symbolic and CNN-based predictors using multiple linear regression on accuracy averaged across subjects and trials for each shape pair(1). For human groups, accuracy was first averaged across individuals and trials ($\approx$ 40 shape–intruder types), and regression coefficients were then estimated at the group level. For monkeys, regressions were conducted separately for each individual using accuracy averaged across trials for each shape pair. The dependent variable represents the proportion of correct responses for that stimulus pair. This analytic approach is non-ideal in several respects.

First, the aggregation collapses across both trials and individuals before model estimation and therefore loses information about the statistical confidence/certainty behind each data point. For example, 7/10 and 70/100 trials would both be aggregated to 0.70, but you would have considerably less certainty about the former (e.g. CI: 0.35-0.9) than the latter (e.g. CI: 0.6-0.78). The inferences that are made about groups should be sensitive to the reliability with which these averages are measured, and are in our Bayesian analysis.

Second, their approach treats the summary statistic as the key outcome rather than modeling the underlying decision process–in particular, the analysis on accuracy does not take into account the "pull" of different alternatives. For example, some trials might have more close competitors to the target. Our analysis model captures the actual choices made (as opposed to just whether those choices were correct or not) which is important when there are multiple options. We use a predictor for the probability of choosing each shown item, and model choice as a softmax function of those predictors. To illustrate, in an item where there are, say 3 close competitors to the target according to the predictors and regression weights, our approach would predict a 1/(3+1) = 25% chance of responding correctly because there are four options (a target and 3 close alternatives). Sablé-Meyer et al.(2021)'s model won't in general ever predict less than 50%, which it could achieve by ignoring all the predictors. Theirs can do this because it doesn't have to choose the right answer, it only has to say whether or not the trial was correct(1). In this way—because it is not a model of how the choice is made out of all the options–success is artificially inflated (As discussed below, we use our technique to re-analyze their data). Our approach provides a more direct test of how representations influence decisions because the representations of each individual item are modeled and incorporated into the statistics.

Additionally, our analysis is focused on reliably estimating parameters for individual participants, allowing us to look at the overlap between distributions individual-by-individual. This is critical for the claim of human uniqueness, because one should predict that no non-human primates overlap in parameter values with any human, something that Sablé-Meyer at al. did not evaluate. Our goal was to assess whether high-level perceptual (IT) and symbolic representations coexist within individuals and overlap across species. Importantly, overlap in parameters between monkeys and humans reflects intermixing of individual-level representational profiles. Such overlap would not be detectable in analyses that collapse behavior across subjects and trials.

Finally, our regression technique has the additional advantage that it allows us to evaluate the contribution of predictors, while rigorously controlling for uncertainty in the contribution of other variables, something which is important when examining the effect of partially correlated predictors.

B. Evidence of Symbolic Sensitivity in Monkeys. While they do not use this as evidence of symbolic representations, Sablé-Meyer et al. (2021)'s data did show moderate correlations between the symbolic model and monkeys' behavior ($r \approx .26$–$.63$; mean $\approx .45$; see values from their Fig. 4C (Figure S12 below))(1). We found that those symbolic effects in monkeys hold in multivariate analyses of trial-level data that simultaneously model Symbolic and IT predictors. In these trial-level analyses of the Sablé-Meyer monkey data, symbolic effects emerged at the group level and for most individual baboons (8 of 11). Thus, symbolic sensitivity is detectable in the Sablé-Meyer et al. (2021) monkey data when individual trials rather than aggregated data are analyzed (see Figure S13 for results).

C. Predictive Performance. To more directly compare frameworks, we evaluated the ability of both models to predict above chance. Sablé-Meyer's regression predicts group-level accuracy (chance = 0.5). Our Bayesian model predicts trial-level choice among six alternatives (chance = 0.17). As a result, the two statistical models are computing on different data and cannot be compared using standard measures like AIC or BIC (which require models to predict the same data). Instead, a simple way to compare these different baselines is to compute the proportion from chance each model is, yielding a measure of the relative performance-above-chance for each model. This is shown in the Figure S14 (left). In this metric, our measure provides substantially more – 2 to 3x – the increment in performance. An alternative is to just look at the overall predictive accuracy (Figure S14, right). This shows that both models are close to 50% accuracy, with the Sablé-Meyer just above. But, note that the Sablé-Meyer model can achieve 50% accuracy just at baseline (i.e. ignoring predictors) since it is predicting two options, whereas ours achieves close to 50% accuracy even though chance is 17%. Thus, ours model is contributing substantially

more predictive information above chance than the Sablé-Meyer model. This is likely due to the fact that our trial-level distance-based formulation provides a more sensitive test of representational contributions.

D. Discussion Summary. In summary, our framework increases sensitivity to heterogeneous and overlapping representations across species and allows detection of mixed IT–symbolic strategies that would be obscured by aggregation with multiple linear regression approaches. Our findings do not contradict the presence of symbolic sensitivity in prior work but demonstrate that such sensitivity exists along a graded continuum shared by monkeys and humans.

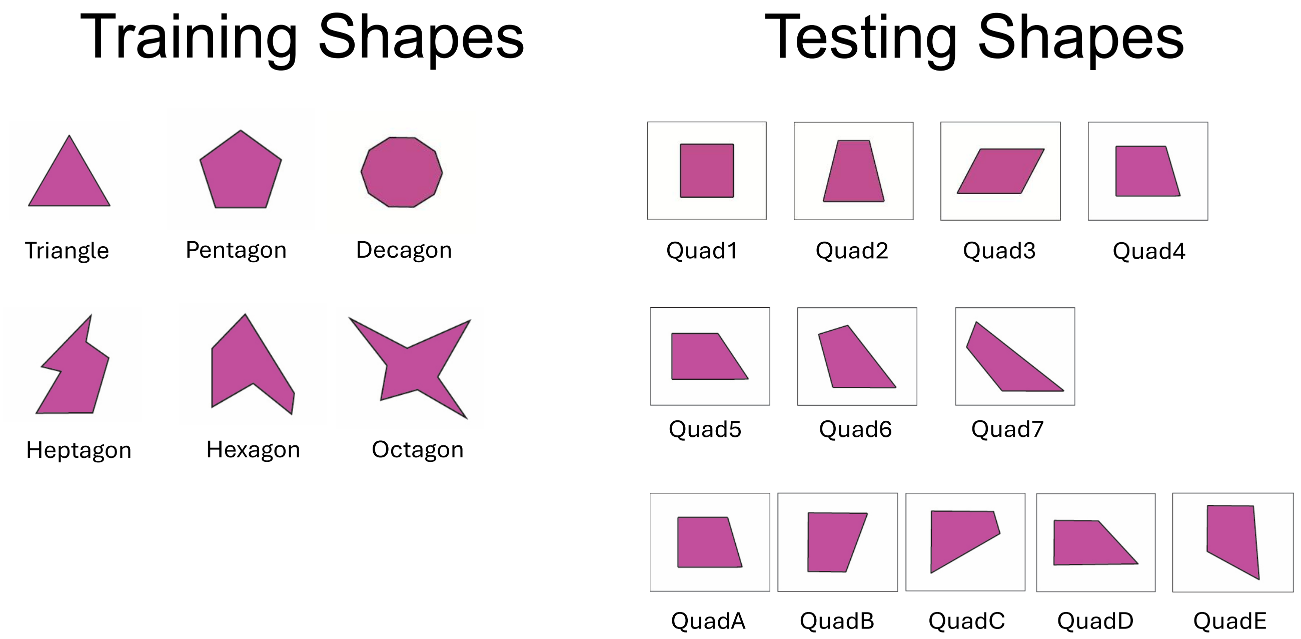

Fig. S1. All stimuli in the Match-to-Sample Task. Figure S1 shows all shapes that we used in the Match-to-Sample Task (Left: Training Shapes; Right: Testing Shapes). In the rotation Match-to-Sample Task, we rotated each shape 20 degrees clockwise and counterclockwise.

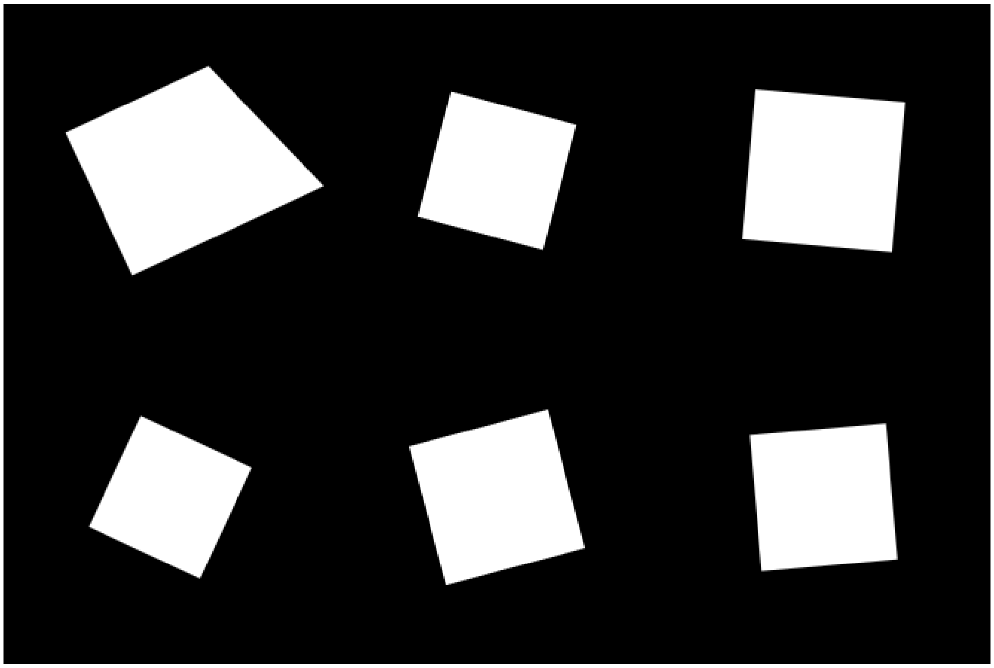

The Intruder Task

Fig. S2. The Intruder Task Illustration.Figure S2 illustrates the Intruder Task used by Sablé-Meyer et al. (2021)(1). Participants were required to identify the deviant shape among five other shapes that varied in size and rotation.

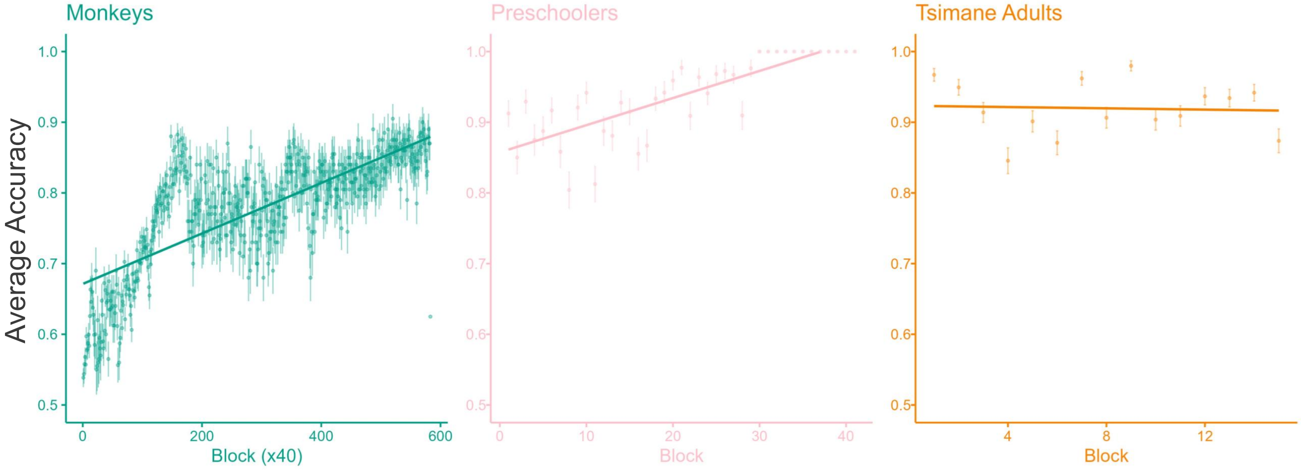

Fig. S3. Training Accuracy across Groups in the Match-to-Sample Task. Figure S3 shows the training accuracy over sessions in monkeys, preschoolers, and adults. All subjects showed accuracy above 70% in the Match-to-Sample Task after training. Humans showed a relatively high accuracy, while monkeys started around chance level but approached the human accuracy after training. The x-axis represents the number of training block, and the y-axis represents the average accuracy of each block. Each dot represents the accuracy of a single training block. Each block contains 5 trials for human groups and 200 trials for monkeys.

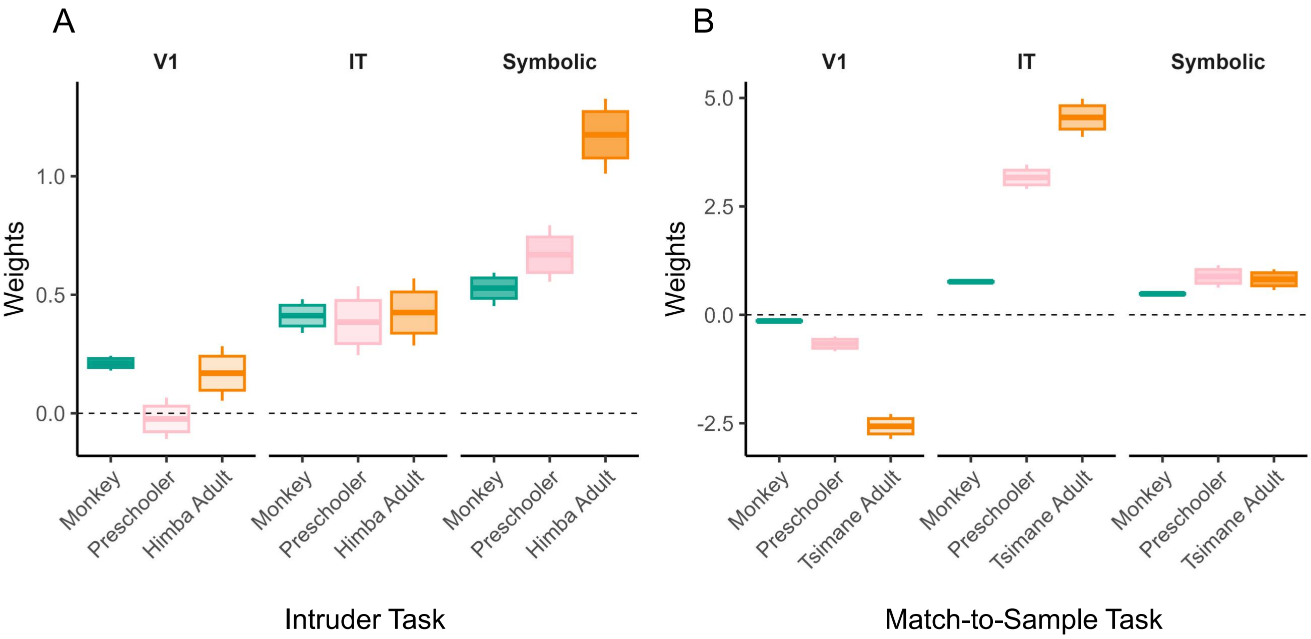

Fig. S4. Group-level Bayesian Analysis without Non-linear transformation – V1-IT-Symbolic Model. Figure S4 plots the Bayesian weights for a V1-IT-Symbolic Bayesian model without any non-linear transformation parameters in the Intruder Task (Left) and the Match-to-Sample Task (Right). The x-axis represents the groups (Monkeys, Preschoolers, Himba/Tsimane Adults), and the y-axis represents the standardized weight estimated from the Bayesian model. Each box-and-whisker marks the posterior mean, ± 1 SD, and the whiskers denote the 90% highest density interval (HDI). This result supports that our V1-IT-Symbolic model is robust regardless of the non-linear transformation.

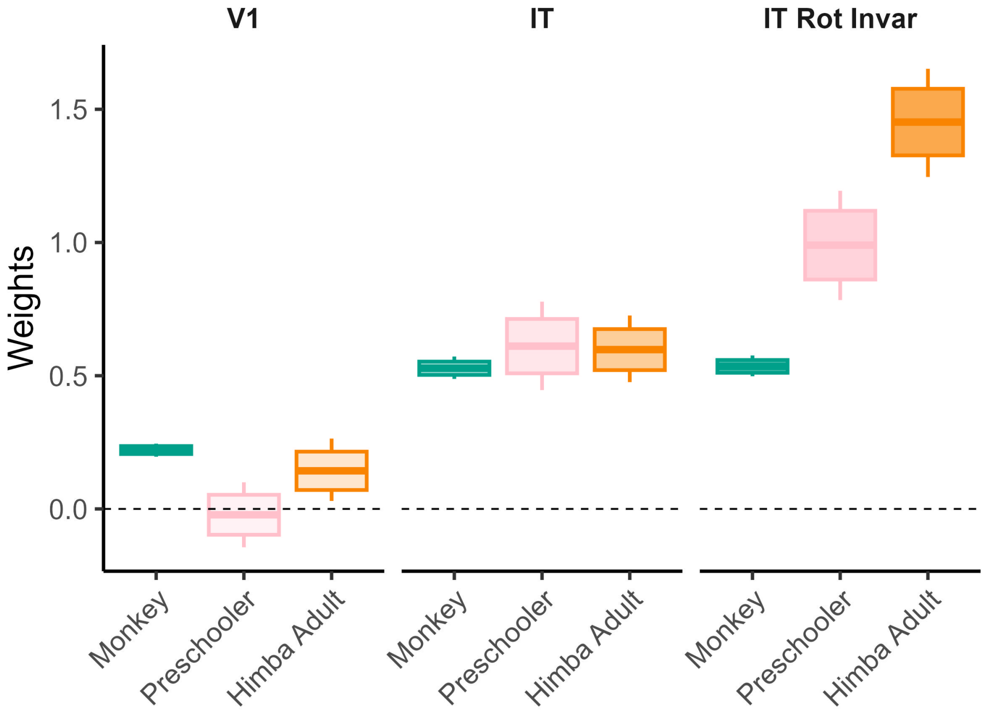

Fig. S5. Group-level Bayesian Analysis without Non-linear transformation – V1-IT-IT Rot Inv Model. Figure S5 plots the Bayesian weights for a V1-IT-IT Rot Invar Bayesian model without any non-linear transformation parameters. The x-axis represents the groups (Monkeys, Preschoolers, Himba adults), and the y-axis represents the standardized weight estimated from the Bayesian model. Each box-and-whisker marks the posterior mean, ± 1 SD, and the whiskers denote the 90% highest density interval (HDI). This result supports that our V1-IT-IT Rot Invar model in the Intruder Task is robust regardless of the non-linear transformation.

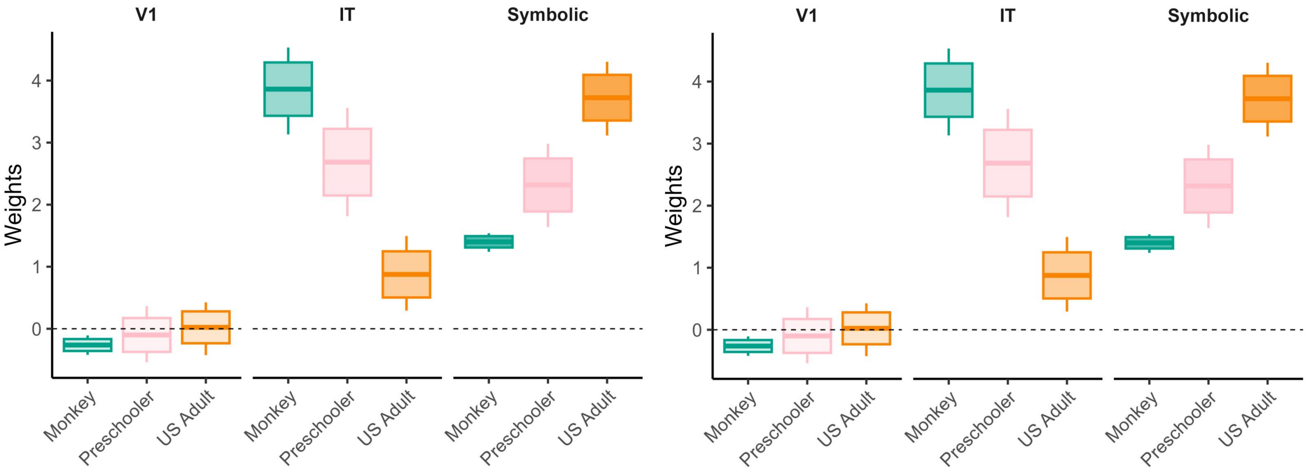

Fig. S6. Group-level Bayesian Analysis without Non-linear transformation – V1-IT-Symbolic Model (Rotated Match-to-Sample Task). Figure S6 plots the Bayesian weights for a V1-IT-Symbolic Bayesian model with (left) or without non-linearities (right) in the Rotated Match-to-Sample Task. The x-axis represents the groups (monkeys, preschoolers, US adults), and the y-axis represents the standardized weight estimated from the Bayesian model. Each box-and-whisker marks the posterior mean, ± 1 SD, and the whiskers denote the 90% highest density interval (HDI). This result reconfirms that our V1-IT-Symbolic model in the Rotated Match-to-Sample Task is robust regardless of the non-linear transformation.

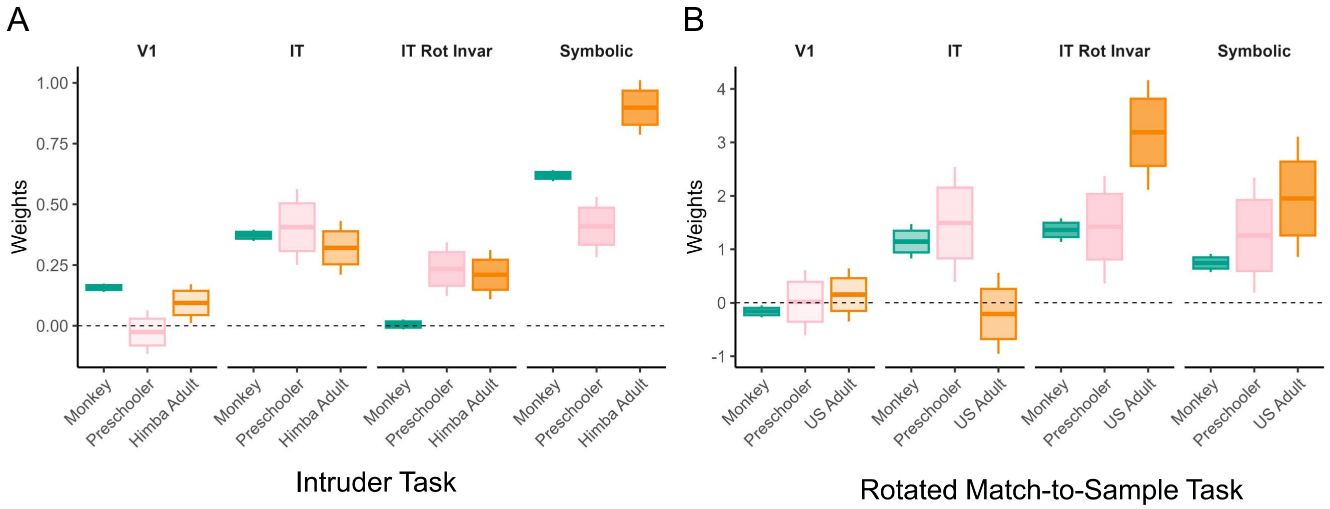

Fig. S7. Group-level Bayesian Analysis with V1, IT, IT Rot Inv, Symbolic. Figure S7 plots the Bayesian weights for the V1-IT-IT Rot Inv-Symbolic Model in the Intruder Task and the Match-to-Sample Task when shapes were rotated. The x-axis represents the groups (Monkeys, Preschoolers, Himba/US Adults), and the y-axis represents the standardized weight estimated from the Bayesian model. Each box-and-whisker marks the posterior mean, ± 1 SD, and the whiskers denote the 90% highest density interval (HDI).

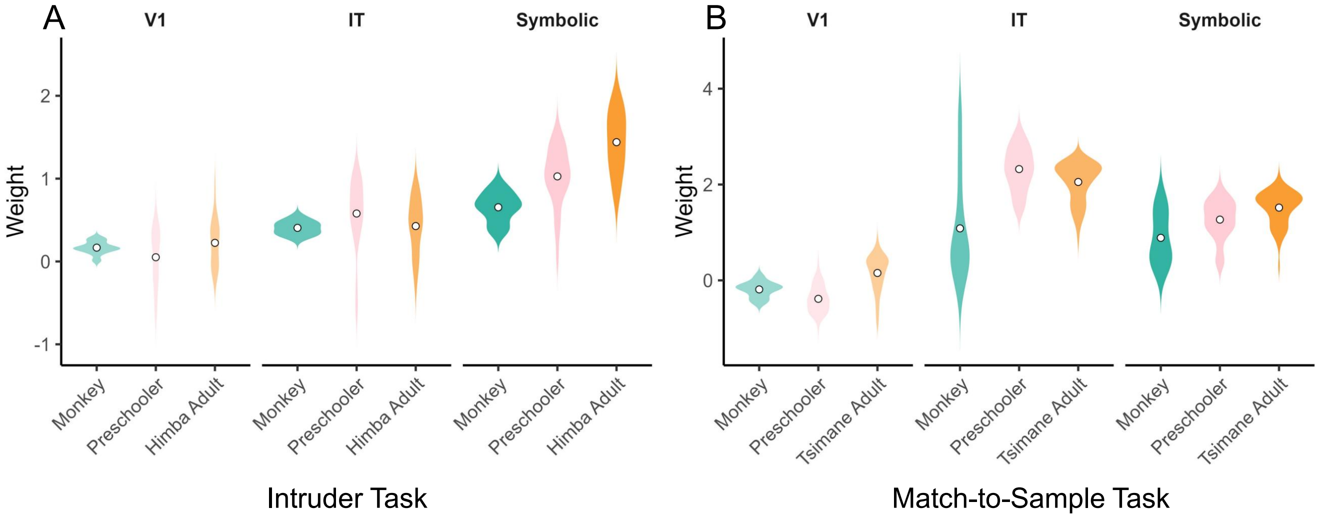

Fig. S8. Bayesian weights of V1-IT-Symbolic Model at the Participant-Level. Fig S8 plots the participant-level Bayesian weights for the V1-IT-Symbolic model in both the Intruder Task (Left) and the Match-to-Sample Task (Right). The x-axis represents the groups (Monkey, Preschooler, Tsimane/Himba adults), and the y-axis represents the standardized weight estimated from the Bayesian model. Each violin marks the posterior mean. The participant-level Bayesian weights for the V1-IT-Symbolic model mirrors the group-level Bayesian weights.

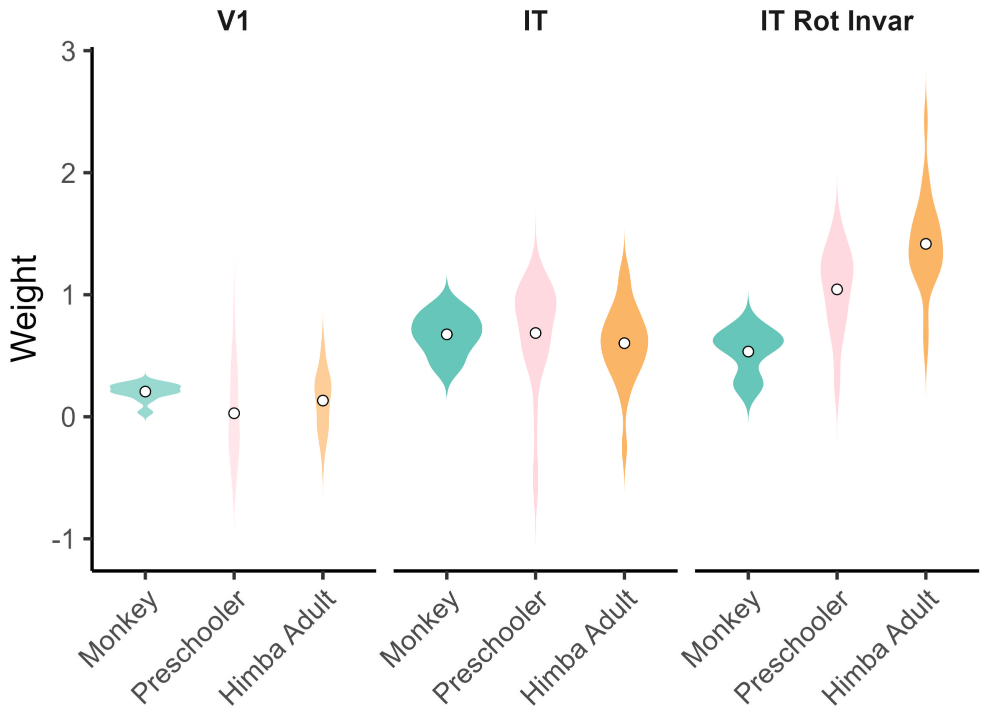

Fig. S9. Bayesian weights of V1-IT-IT Rot Invar Model at the Participant-Level. Fig S9 plots the participant-level Bayesian weights for V1-IT-Rot Invar model in the Intruder Task, The x-axis represents the groups (Monkey, Preschooler, Himba adults), and the y-axis represents the standardized weight estimated from the Bayesian model. Each violin marks the posterior mean. The participant-level Bayesian weights for the V1-IT-Rot Invar model mirrors the group-level Bayesian weights.

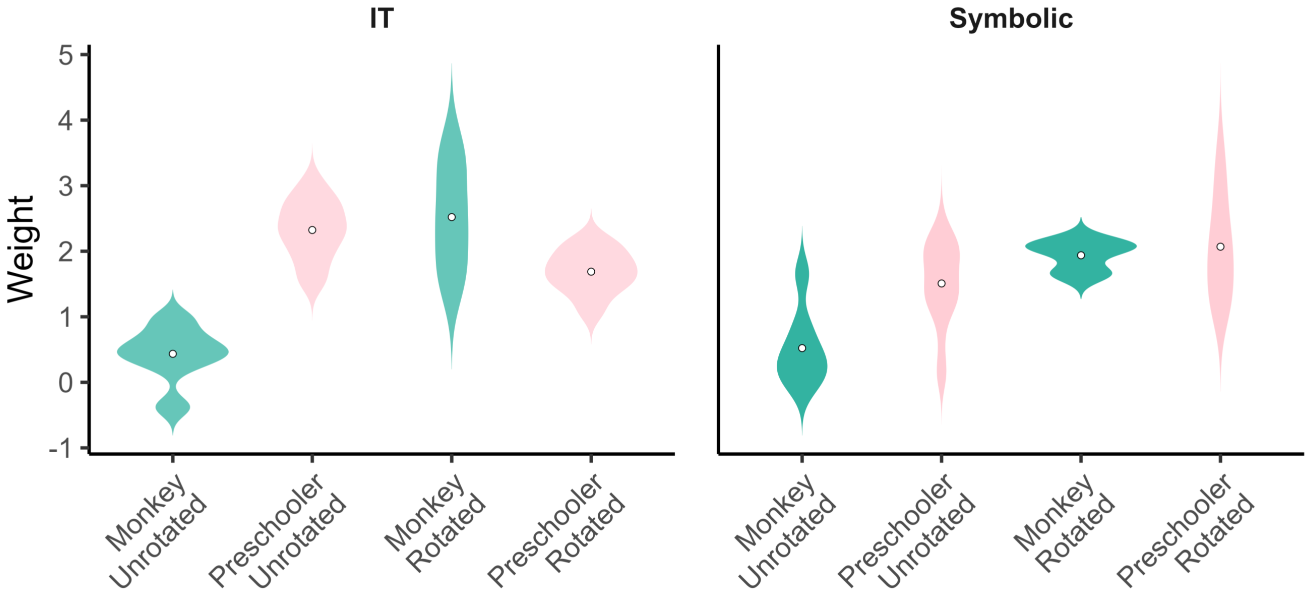

Fig. S10. Participant-Level Bayesian weights of V1-IT-Symbolic Model in the Rotated and Normal Match-to-Sample. Correlation among IT, IT Rot Inv, Symbolic. Fig S10 plots the participant-level Bayesian IT and symbolic weights for V1-IT-Symbolic model in the Rotated Match-to-Sample Task. The x-axis represents the participant group (monkeys, preschoolers), and the y-axis represents the standardized weight estimated from the Bayesian model. Each violin marks the posterior mean. The participant-level Bayesian IT and symbolic weights mirror the group-level Bayesian IT and symbolic weights.

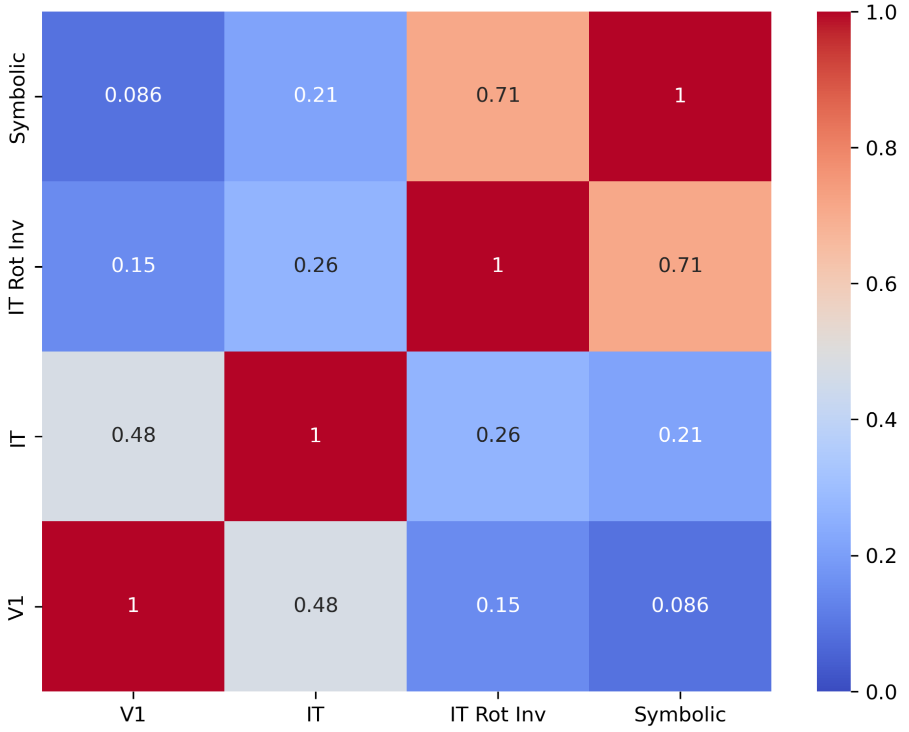

Fig. S11. Correlation among V1, IT, IT Rot Inv, Symbolic. Figure S11 shows the Pearson correlation between V1 representation, IT representation, IT Rotation Invariant representation, and Symbolic representation in the Intruder Task. The Symbolic and IT Rotation Invariance have a high correlation of 0.71, a much higher correlation than IT and IT Rotation Invariance (0.26), which shows that the symbolic representation is specifically related to rotational invariant shape representation.

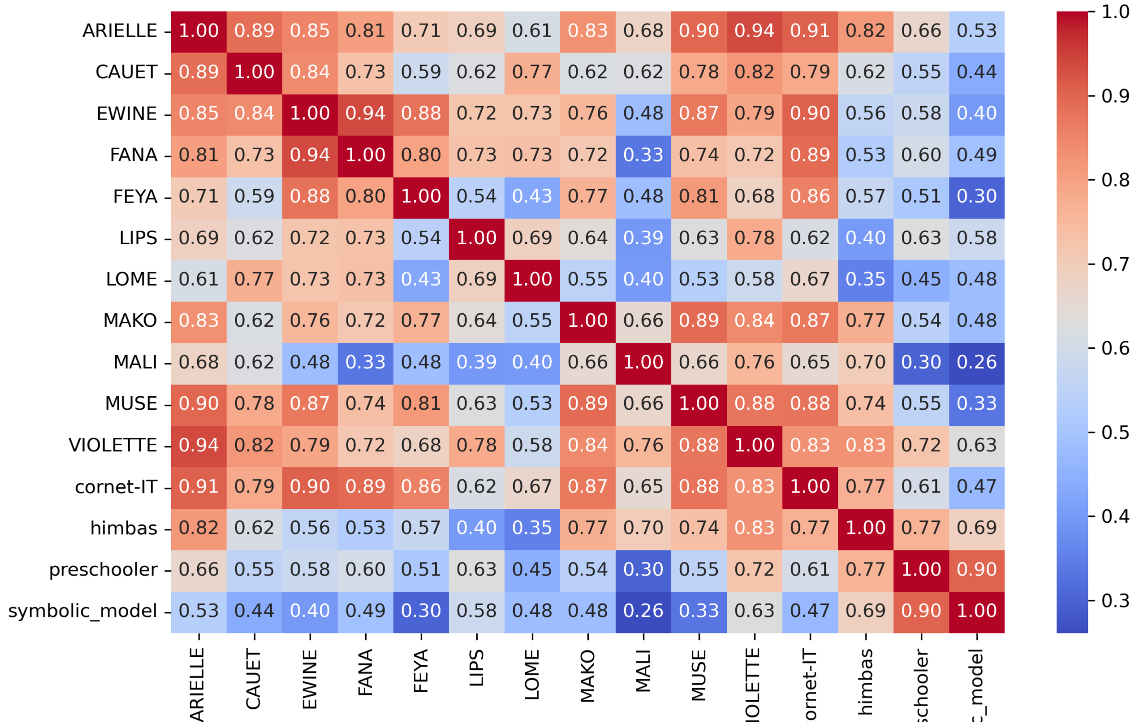

Fig. S12. Correlation among baboons, human groups, and the symbolic model (Adapted from Figure 4C in Sablé-Meyer et al (2021). Figure S12 shows the Pearson correlation between the performance of individual baboons, human groups, and the symbolic model. Monkeys show moderate correlations with the symbolic model($r \approx .26$–$.63$; mean $\approx .45$), which is consistent with the observed symbolic effect under trial-level analysis.

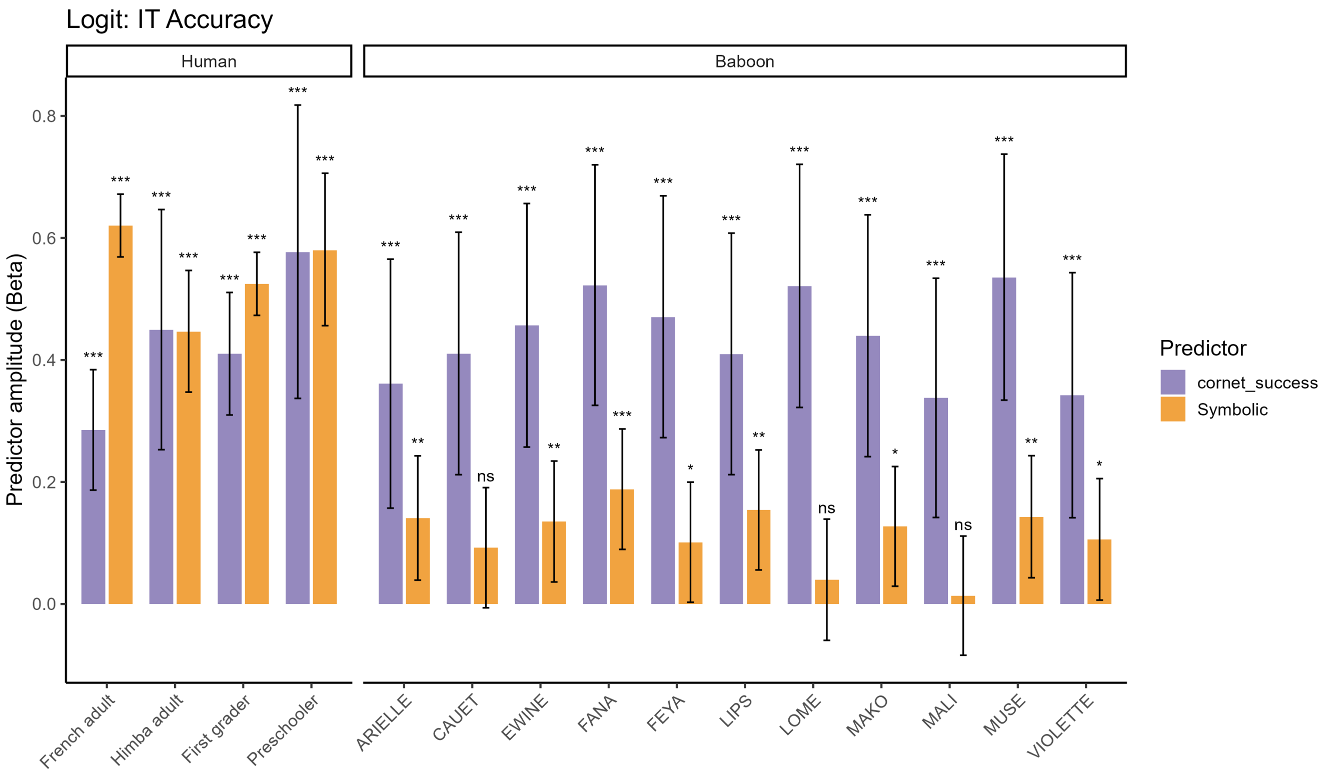

Fig. S13. Detectable symbolic effect in most of baboons and human groups at the trial-level analysis with the same predictors in Sablé-Meyer et al. (2021). Fig S13 plots the standardized Beta of predictors (IT accuracy and Symbolic distance) in a logistic regression model at the trial-level analysis. X-axis represents the group name and the y-axis represents the predictor amplitude (IT and Symbolic). The symbolic distance contributes significantly across most of the baboons (8 out of 11) and all human groups, which reveals that the symbolic effect is detectable in monkeys in the Intuder Task. These findings show that symbolic effects are detectable in the Sablé-Meyer data using the same predictors they used (IT accuracy and Symbolic distance) just by looking at trial-level data for all groups. However, as we describe in Supplemental Discussion, their IT accuracy predictor is imprecise and a more sensitive predictor is IT distance – which we used in our analyses in the main article.

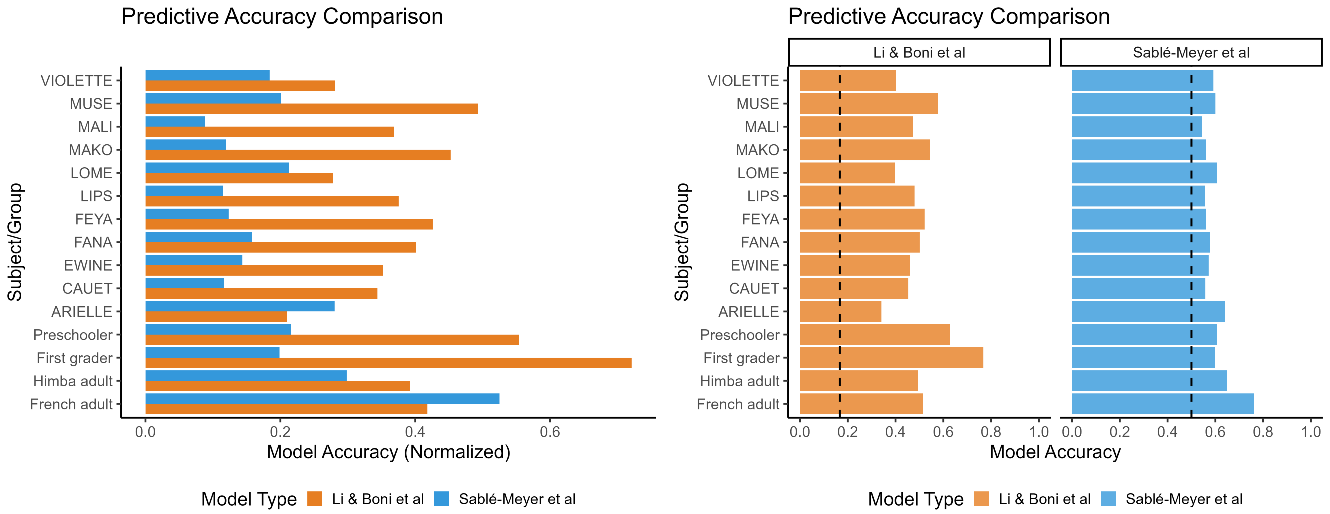

Fig. S14. Fig S14 compares the trial-level predictive accuracy between the current Bayesian model (chance = 17%) and the Sablé-Meyer et al. (2021) regression model (chance = 50%). The x-axis illustrates both normalized predictive accuracy adjusted for baseline chance (left) and raw predictive accuracy (right). The y-axis represents the predictor amplitude (IT and Symbolic). The Bayesian regression (Li & Boni et al) contributed more predictive information than Sablé-Meyer et al. (2021) 's regression.

References

1. M Sablé-Meyer, et al., Sensitivity to geometric shape regularity in humans and baboons: A putative signature of human singularity. *Proc. Natl. Acad. Sci.* **118**, e2023123118 (2021).
